# Association of Visual Acuity and Cognitive Impairment in Older Individuals: Fujiwara-kyo Eye Study

**DOI:** 10.1089/biores.2016.0023

**Published:** 2016-08-01

**Authors:** Masashi Mine, Kimie Miyata, Masayuki Morikawa, Tomo Nishi, Nozomi Okamoto, Ryo Kawasaki, Hidetoshi Yamashita, Norio Kurumatani, Nahoko Ogata

**Affiliations:** ^1^ Department of Ophthalmology, Nara Medical University, Kashihara, Nara, Japan.; ^2^ Mie Prefectural Mental Care Center, Tsu, Mie, Japan.; ^3^ Department of Community Health and Epidemiology, Nara Medical University, Kashihara, Nara, Japan.; ^4^ Department of Public Health, Graduate School of Medical Science, Yamagata University, Yamagata, Japan.; ^5^ Department of Ophthalmology and Visual Science, Yamagata University Faculty of Medicine, Yamagata, Japan.

**Keywords:** aging, neuroscience, regeneration

## Abstract

Both visual impairment and cognitive impairment are essential factors that determine the quality of life in the aged population. The aim of this study was to determine if a correlation existed between visual acuity and cognitive impairment in an elderly Japanese population. The Fujiwara-kyo Eye Study was a cross-sectional study of individuals aged ≥68 years who lived in Nara Prefecture of Japan. Participants underwent ophthalmological examinations and cognitive function test. A mild visual impairment was defined as having a best corrected visual acuity (BCVA) >0.2 logarithm of the minimum angle of resolution (logMAR) units in the better eye. Cognitive impairment was defined as having a Mini-Mental State Examination (MMSE) score of ≤23 points. A total to 2818 individuals completed the examinations. The mean age of the participants was 76.3 ± 4.8 years (mean ± standard deviation). The mean BCVA of the better eye was −0.02 ± 0.13 logMAR units and 6.6% subjects were classified as being mildly visually impaired. The mean MMSE score was 27.3 ± 2.3 and 5.7% subjects were classified as being cognitively impaired. The proportion of subjects with cognitive or moderate visual impairment increased with age, and there was a significant correlation between the visual acuity and MMSE score (*r* = −0.10, *p* < 0.0001). Subjects with mild visual impairments had 2.4 times higher odds of having cognitive impairment than those without visual impairment (odds ratio 2.4, 95% confidence interval, 1.5–3.8, *p* < 0.001) after adjusting for age, sex, and length of education. We conclude that it may be important to maintain good visual acuity to reduce the risk of having cognitive impairment.

## Introduction

The proportion of older individuals in the population has been rapidly increasing globally. In developed countries, the proportion of the population ≥65 years was 16% in 2010, and it has been estimated that the proportion will increase to 27% in 2060.^1^ Japan is considered to be the most aged country in the world and the Japanese Cabinet Office has reported that the nationwide proportion of individuals ≥65 years old was 23%, and that of ≥75 years old was 11% in 2010.^2^ In 2060, these numbers are estimated to increase to 40% and 27%, respectively. In such an aged society, it is important that older individuals maintain their health and have a good quality of life (QOL) even at a later stage of life when various age-related functional and physical disorders develop.

Both visual and cognitive impairments are essential factors that limit the activities of daily living and the QOL. According to the Japanese Ministry of Health, Labour and Welfare, the prevalence of dementia in persons aged ≥65 years was estimated to be 15% in 2010.^3^ Iwano et al. reported that the prevalence of vision impairments, defined as a having corrected Snellen visual acuity of <10/20 in the better eye, was 5.6% in subjects aged 70 to 79 years in 2004.^4^ They also reported that the risk of developing visual impairments increased by 3.9-fold for every 10-year increase in age.

Earlier studies^5–8^ and population-based epidemiological studies involving older individuals^9–14^ have shown that there is an association between visual impairment and cognitive function. Such findings have been reported by the Berlin Aging Study,^9^ Australian Longitudinal Study of Aging,^10,11^ Maastricht Aging Study,^12^ Leiden 85+ Study,^13^ and Blue Mountains Eye Study.^14^ However, there are still contradictions in linking visual impairment and cognitive function.^15,16^

To date, there has been no study reporting the link between visual impairment and cognitive function in Japan.^17–19^ Because Japan has the longest life span in the world,^20^ it is important to determine whether there is a significant correlation between visual impairment and cognitive function in the elderly Japanese. Thus, the purpose of this study is to determine whether there is an association between visual acuity and cognitive function in an elderly Japanese population.

## Materials and Methods

### Participants

The Fujiwara-kyo Study was a cohort study conducted in Nara and undertaken to investigate the functional capacity and the QOL of a community of elderly population.^21,22^ Study participants were ≥65 years, living in their own home, and residents of Nara Prefecture. The participants were volunteers who responded to our recruitment announcements. The Fujiwara-kyo Study was initially performed in 2007 with plans to do follow-up examinations every 5 years. The ophthalmological survey (Fujiwara-kyo Eye Study) was begun at the second stage in February to November 2012. Therefore, most of the individuals were ≥70 years because they were recruited at 2007 when they were 65 years or older, but new subjects aged ≥65 years (61 individuals) were also recruited during this second stage. We used data from the examinations done in 2012 as a cross-sectional study.

All of the subjects underwent a basic interview to obtain their sociodemographic data, their general medical condition, and treatment histories. They also underwent systemic examinations, including physical anthropometric assessments, physical fitness assessments, and blood tests. These surveys were conducted in accordance with the tenets of the Declaration of Helsinki, and the protocol was approved by the Ethics Review Board of Nara Medical University. A signed informed consent form was obtained from all participants.

### Ophthalmological examinations

The uncorrected and corrected visual acuities of both eyes were measured with a Landolt ring chart at 5 m with correction of any refractive errors determined by an automatic refractometer and keratometer (ARK-700A; Nidek, Aichi, Japan). The visual acuity was measured according to the standard of the International Organization for Standardization.^23^ The decimal best-corrected visual acuity (BCVA) of the better eye was converted to the logarithm of the minimum angle of resolution (logMAR) units for statistical analyses. The degree of visual impairment was classified as recommended by the International Council of Ophthalmology report^24^ and the Japanese driver's license requirement on vision. Thus, subjects with BCVA of >0.2 logMAR units in the better eye were classified as being mildly visually impaired.

### Cognitive function test

The Mini-Mental State Examination (MMSE) was used for the cognitive function test. This test was developed by Folstein et al. in 1975 and is commonly used for dementia screening.^25^ It consists of five downstream items of orientation, memory, attentiveness for calculations, speech function, and design capacity. This test was performed by verbal questioning of 5- to 10-min duration by skilled clinical psychologists. The maximum score for the MMSE is 30 points, and individuals with a score of ≤23 points were classified as having cognitive impairments. We also analyzed the MMSE excluding the following vision-related five items: “naming two objects,” “following a 3-step command,” “reading and following instruction,” “writing a sentence,” and “visual reconstruction” and the maximum score for this was 22 points. This was conducted because Reischies and Geiselmann^5^ and Busse et al.^8^ reported that these five items of the MMSE were dependent on the visual acuity.

### Statistical analyses

The differences in the sociodemographic and health characteristics of individuals with and without mild visual impairments were analyzed by the chi-square and unpaired *t*-tests. The correlations between the BCVA of the better eye and the MMSE score were determined by Pearson's correlation coefficient. A scatter plot was made with the visual acuity in logMAR units plotted on the abscissa and the MMSE scores plotted on the ordinate. The best fit regression line was calculated for these data. The differences of mean MMSE scores between individuals with or without mild visual impairment were analyzed by analysis of covariance (ANCOVA) with adjustments for age, sex, length of education, and history of stroke. Multiple logistic regression analyses were used to determine the association between visual and cognitive impairment with adjustments for age, sex, length of education, and history of stroke. The odds ratios (OR) and 95% confidence intervals (CI) for having cognitive impairment in terms of visual acuity were calculated. Statistical analyses were performed with the SPSS (version 22.0; SPSS, Inc., Chicago, IL). Two-tailed *p*-values were used in all of the analyses. A *p*-value <0.05 was taken to be significant.

## Results

There were 2873 individuals who participated in the Fujiwara-kyo Eye Study. Of these, 2818 individuals completed all of the examinations (98.1%) and were used in the statistical analyses. Fifty-five participants were excluded because the vision or cognitive function tests were not performed or were incomplete.

The age of the 2818 participants ranged from 68 to 100 years with a mean of 76.3 ± 4.8 years (±standard deviation [SD]) (Table 1). There were 1486 (52.7%) men and 1332 women. There were 1166 subjects whose age was ≤74 years, 965 subjects aged 75 to 79 years, 494 subjects aged 80 to 84 years, 166 subjects aged 85 to 90 years, and 27 subjects aged ≥90 years. There were 2172 (77.1%) subjects whose level of education was ≤12 years, and there were 646 (22.9%) subjects whose level of education was ≥13 years (Table 1).

**Table 1.** **Distribution of Characteristics of the Participants (*n* = 2818)**

Mean age ± SD (range), years	76.3 ± 4.8 (68–100)
Men/women, *n* (%)	1486 (52.7)/1332 (47.3)
Mean BCVA in logMAR units in the better eye ± SD (range)	−0.02 ± 0.13 (−0.30–1.39)
Mild visual impairment (%)	187 (6.6)
Mean MMSE score ± SD (range)	27.3 ± 2.3 (14–30)
Cognitive impairment (%)	160 (5.7)
Mean MMSE score excluding 5 vision-related items^a^ ± SD (range)	19.4 ± 2.2 (8–22)
Length of education with less than 12 years (%)	2172 (77.1)

MMSE score ranged from 0 to 30. MMSE score excluding five items ranged from 0 to 22. Cognitive impairment defined as MMSE score ≤23. Mild visual impairment defined as logMAR units >0.2 in the better eye.

^a^ Vision-related five items: “naming two objects,” “following a 3-step command,” “reading and following instruction,” “writing a sentence,” and “visual reconstruction.”

BCVA, best corrected visual acuity; logMAR, logarithm of the minimum angle of resolution; MMSE, Mini-Metal State Examination; SD, standard deviation.

The BCVA of the better eye ranged from −0.30 to 1.39 logMAR units with a mean of −0.02 ± 0.13 logMAR units. Of the 2818 participants, 187 (6.6%) were classified as being mildly visually impaired, that is, having BCVA >0.2 logMAR units in the better eye (Table 1).

The MMSE scores ranged from 14 to 30 points with a mean of 27.3 ± 2.3. Of the 2818 participants, 160 individuals (5.7%) were classified as being cognitively impaired; that is, MMSE score ≤23 points. The MMSE scores excluding five vision-related items with a maximum score of 22 points ranged from 8 to 22 points with a mean of 19.4 ± 2.2 (Table 1).

The number of subjects with mild visual impairment was 45 (3.9%) of 1166 subjects in the age ≤74 years, 68 (7.0%) of 965 subjects in the age 75 to 79 years, 44 (8.9%) of 494 subjects in the age 80 to 84 years, 22 (13.3%) of 166 subjects in the age 85 to 90 years, and 8 (29.6%) of 27 subjects in the age ≥90-year groups. The number of subjects with cognitive impairment was 36 (3.1%) of 1166 subjects in the age ≤74 years, 55 (5.7%) of 965 subjects in the age 75 to 79 years, 44 (8.9%) of 494 subjects in the age 80 to 84 years, 18 (10.8%) of 166 subjects in the age 85 to 90 years, and 7 (28.9%) of 27 subjects in the age ≥90-year groups. The proportion of subjects with cognitive or mild visual impairment increased with increasing age.

The mean age of the 160 subjects with cognitive impairment was 79.2 ± 5.4 years, and that of the 2658 subjects without cognitive impairment was 76.1 ± 4.7 years (Table 2). The mean age of the 187 subjects (6.6%) with mild visual impairment was 78.7 ± 5.7 years, and that of 2631 subjects (93.4%) without mild visual impairment was 76.1 ± 4.7 years. The age of subjects in whom the cognitive function or visual acuity was classified as being impaired was significantly older than those without the impairments (*p* < 0.001) (Table 2).

**Table 2.** **Sociodemographic and Health Characteristics of Participants with Cognitive Impairment and Mild Visual Impairment**

	Cognitive impairment			Mild visual impairment		
Factors	Absent (*n* = 2658)	Present (*n* = 160)	*p*	Absent (*n* = 2631)	Present (*n* = 187)	*p*
Mean age ± SD, years	76.1 ± 4.7	79.2 ± 5.4	<0.001	76.1 ± 4.7	78.7 ± 5.7	<0.001
Men, %	52.4	58.8	0.116	53.7	38.5	<0.001
Length of education, less than 12 years, %	76.5	86.3	0.004	76.6	84.0	0.021
History of disease, %						
Stroke	6.0	5.0	0.447	5.9	9.6	0.037
Cerebral infarction	4.7	5.0	0.863	4.5	7.5	0.065
Angina	8.3	8.1	0.931	8.4	6.4	0.332
Myocardial infarction	2.7	3.1	0.779	2.8	2.1	0.586
Diabetes mellitus	14.8	16.3	0.605	14.8	16.0	0.632
Hypertension	46.0	39.5	0.118	45.2	50.8	0.143
Hypercholesterolemia	26.1	19.4	0.059	26.1	20.9	0.116
Current smoking	5.6	9.0	0.075	5.8	5.5	0.845

*p*-Values are for chi-square tests or unpaired *t*-tests performed. Cognitive impairment defined as MMSE score ≤23. Mild visual impairment defined as logMAR units >0.2 in the better eye.

Cognitive impairments were significantly associated with age and length of education. Mild visual impairments were significantly associated with age, sex, length of education, and a history of stroke (Table 2).

**FIG. 1.**
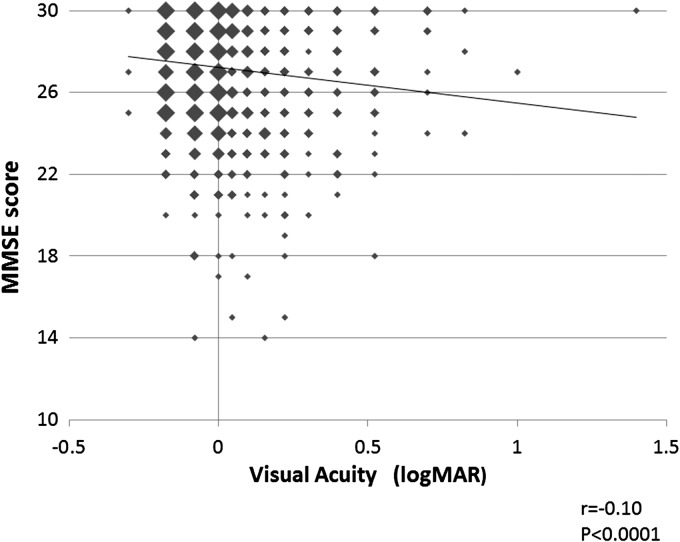
Relationship between visual acuity and MMSE score. The MMSE score is significantly correlated with the BCVA of the better eye (*r* = −0.10, *p* < 0.0001). BCVA, best corrected visual acuity; MMSE, Mini-Mental State Examination.

The correlation between the BCVA of the better eye and the MMSE score was significant (*r* = −0.10, *p* < 0.0001, Pearson's correlation coefficient, Fig. 1).

The mean MMSE score in subjects with mild visual impairments was significantly lower than in those without mild visual impairments after adjusting for age, sex, length of education, and history of stroke (26.8 vs. 27.3, *p* < 0.05, Table 3). After excluding the five vision-related items in the MMSE, the significant association between the MMSE score and the presence of mild visual impairments remained after adjusting for age, sex, length of education, and history of stroke (19.1 vs.19.4, *p* < 0.05, Table 3).

**Table 3.** **Adjusted Mean MMSE Score (Standard Error) by the Presence or Absence of Mild Vision Impairment**

		Mean MMSE score (standard error)			
		Mild visual impairment			
MMSE items	Range of MMSE scores	Absent	Present	F statistic	*p*
All items	0–30	27.3 (0.05)	26.8 (0.17)	6.359	0.012
Excluding vision-related 5 items	0–22	19.4 (0.04)	19.1 (0.16)	4.287	0.038

ANCOVA adjusted for age, sex, length of education, and history of stroke. Mild visual impairment defined as logMAR units >0.2 in the better eye.

The subjects were classified into four groups by the BCVA, and the prevalence of cognitive impairment was significantly higher in the group with poorer visual acuity (Table 4). The prevalence of cognitive impairment in subjects with BCVA of 0 to 0.1 logMAR units in the better eye was twofold higher than in subjects with BCVA of ≤0 logMAR units (8.0% vs. 4.5%). In addition, compared to subjects with BCVA of ≤0 logMAR units, the OR for cognitive impairment increased to 1.8 (1.2–2.7, 95% CI, *p* = 0.007) in subjects with BCVA of 0 to 0.1 logMAR units and to 3.3 (2.1–5.4, 95% CI, *p* = 0.005) in subjects with BCVA of >0.2 logMAR units after adjusting for age, sex, length of education, and history of stroke (Table 4).

**Table 4.** **Associations Between Visual Acuity and Cognitive Impairment**

		Odds ratio (95%CI)			
BCVA in the better eye, logMAR	Cognitive impairment (%)	Unadjusted	*p*	Adjusted^a^	*p*
≤0	96/2135 (4.5)	1.0 (reference)		1.0 (reference)	
0–0.1	31/386 (8.0)	1.9 (1.2–2.8)	0.004	1.8 (1.2–2.7)	0.007
0.1–0.2	8/110 (7.3)	1.9 (0.9–3.8)	0.083	1.7 (0.8–3.5)	0.187
>0.2	25/187 (13.4)	3.3 (2.1–5.2)	<0.001	3.3 (2.1–5.4)	0.005

^a^ Multivariate regression model, adjusting for age, sex, length of education, and history of stroke.

95% CI, 95% confidence interval.

The prevalence of cognitive impairment was higher in subjects with mild visual impairment than in those without mild visual impairment (13.3% vs. 5.1%, *p* < 0.001) (Table 5). When mild visual impairments (>0.2 logMAR) were present, the OR for the presence of cognitive impairment was 2.9 (1.8–4.5, 95% CI, *p* < 0.001). Age and length of education also significantly affected the presence of cognitive impairments (OR = 1.7 per 5 years of age, 95% CI, 1.4–1.9, *p* < 0.001; OR = 1.9 between education ≤12 years, 95% CI, 1.2–3.0, *p* < 0.001, respectively). After adjusting for age, sex, length of education, and history of stroke, mild visual impairment was significantly associated with a higher odds ratio of having cognitive impairment (OR = 2.4, 95% CI, 1.5–3.8, *p* < 0.001, Table 5).

**Table 5.** **Factors Associated with Cognitive Impairment**

		Odds ratio (95% CI)			
Factors	Cognitive impairment (%)	Unadjusted	*p*	Adjusted^a^	*p*
Age (each 5-year increase)		1.7 (1.4–1.9)	<0.001	1.6 (1.4–1.9)	<0.001
Sex					
Men	94/1486 (6.3)	1.0 (reference)		1.0 (reference)	
Women	66/1332 (5.0)	0.8 (0.6–1.1)	0.117	0.7 (0.5–1.0)	0.039
Length of education ≤12 years					
No	22/646 (3.4)	1.0 (reference)		1.0 (reference)	
Yes	138/2172 (6.4)	1.9 (1.2–3.0)	0.005	2.0 (1.2–3.1)	0.005
History of stroke					
No	152/2646 (5.7)	1.0 (reference)		1.0 (reference)	
Yes	8/172 (4.7)	0.8 (0.4–1.5)	0.448	1.0 (0.5–1.8)	0.956
Mild visual impairment					
No	135/2631 (5.1)	1.0 (reference)		1.0 (reference)	
Yes	25/187 (13.3)	2.9 (1.8–4.5)	<0.001	2.4 (1.5–3.8)	<0.001

^a^ Multivariate regression model, adjusting for age, sex, length of education, and history of stroke.

## Discussion

Our results confirmed that the prevalence of both cognitive impairment and visual impairment increases with increasing age. The subjects with visual impairment defined as BCVA of >0.2 logMAR units in the better eye had significantly lower MMSE scores than those without visual impairment. In addition, a significant correlation was found between the MMSE score and the BCVA of the better eye. A lower BCVA was associated with higher prevalence of cognitive impairment, and even a slight visual reduction (0 to 0.1 logMAR units) was associated with 1.8 times higher odds of having a cognitive impairment. Among the subjects with mild visual impairment (>0.2 logMAR units), the odds of having cognitive impairment were 2.4 times higher than those without mild visual impairment after age, sex, length of education, and history of stroke were adjusted.

There have been earlier studies reporting an association between visual impairment and cognitive impairment. The Blue Mountain Eye Study reported a correlation between visual impairment and cognitive impairment.^14^ In addition, the Maastricht Aging Study,^12^ the Leiden 85+ Study,^13^ and the Aging, Demographics, and Memory study^26^ also found the correlation between visual impairment and cognitive impairment. Our results are comparable to these earlier studies. Our new finding is that the association was consistent in an older population whose age was ≥68 years with a mean of 76.3 years with a relatively larger sample number in an epidemiological study of 2818 subjects. This study is the first to report such association in elderly subjects in Japan.

The association between visual impairment and cognitive impairment has not been observed in some studies.^15,16^ We suggest that this inconsistency may be because of the differences in the method of how the visual acuity was measured.^12–14,26^ For example, the vision was obtained by the self-reporting of visual acuity and no vision test was performed in one study.^26^ In another study, the vision was measured without refractive correction or only with their daily used spectacles.^12^ In our study, the visual acuities were measured with the corrective lenses based on the refraction determined by skilled orthoptists. Thus, the visual acuities in our study are likely to be more accurate, which makes the conclusions more reliable.

To evaluate the cognitive functions, it has been pointed out that the visual acuity-dependent factors were included in the cognitive function test.^5,8^ Thus, Diaz et al.^15^ and Killen et al.^16^ used the MMSE excluding vision-related items and reported that there was no significant correlation between visual and cognitive impairments. Therefore, we performed additional analyses for the MMSE excluding the five vision-related items. We found that the significant association between visual impairment and cognitive impairment still remained.

The exact mechanisms for the coexistence of vision and cognitive impairments have not been fully determined. A long-term loss of sensory stimulation caused by visual impairment could contribute to an atrophy of the central nervous system leading to cognitive hypofunction.^9^ Thus, it was hypothesized that the cognitive hypofunction would improve with an improvement of visual function. This hypothesis was tested by evaluating the cognitive function before and after cataract surgery.^27–29^ Prospective studies have showed that there were improvements in the MMSE scores after cataract surgery.^27–29^ We recently found in another cross-sectional study that the prevalence of cognitive impairment was significantly lower in subjects with history of cataract surgery than in subjects without history of cataract surgery, independent of visual acuity.^30^ However, Jefferis et al. reported that cognitive improvement was not significant considering a learning effect and the degree of vision recovery.^31^ In addition, a randomized study reported that there was no significant improvement in the cognitive function after cataract surgery.^32^ Although the progression of dementia can be slowed down, no improvement was achieved.^33,34^

Previous studies have reported visual impairment-related cognitive hypofunction based on longitudinal studies.^11,26^ However, Hong et al. recently reported that there was no significant association between visual impairment and cognitive function in a 10-year follow-up study.^35^ In any case, it may be difficult to demonstrate whether there is a significant relationship between visual acuity and cognitive functions. The association between the visual acuity and cognitive function is still unclear.

The Fujiwara-kyo Study will have follow-up surveys every 5 years until 2027. With longitudinal observations and longer follow-up times, we should be able to clarify the temporality of the relationship between visual impairment and cognitive impairment.

Our study was a cross-sectional study and some limitations are inherent. The participants were volunteers, which could potentially cause selection bias. The underlying diseases of visual impairment were unidentified and general medical conditions were obtained only by self-reporting. In addition, we used a very minimal definition of visual impairment (Snellen = 20/32) without consideration of visual fields or contrast sensitivity. They may represent a healthier population with better visual acuity and cognitive function than the general population. Actually, only 5.7% of subjects in our study were classified as being cognitive impaired, while the prevalence of cognitively impaired in individuals aged ≥65 years has been estimated to be 15% in Japan.^3^ Similarly, participants of this study may have had better visual acuity (mean of −0.02 ± 0.13 logMAR) than that of the general population. External validity of this study needs to be considered with careful interpretation. Further studies are needed to determine the temporal associations and to determine if an intervention to maintain or improve the visual acuity can delay or prevent the development of cognitive impairment.

In conclusion, we found a significant association between visual and cognitive impairment. We also found that even mild visual impairment was significantly associated with cognitive impairment. Thus, maintaining good visual acuity might decrease the risk of having cognitive impairment.

## Abbreviations Used

ANCOVA: analysis of covariance
BCVA: best corrected visual acuity
CI: confidence intervals
logMAR: logarithm of the minimum angle of resolution
MMSE: Mini-Mental State Examination
OR: odds ratios
QOL: quality of life
